# Research progress on the anticarcinogenic actions and mechanisms of ellagic acid

**DOI:** 10.7497/j.issn.2095-3941.2014.02.004

**Published:** 2014-06

**Authors:** Hong-Mei Zhang, Lei Zhao, Hao Li, Hao Xu, Wen-Wen Chen, Lin Tao

**Affiliations:** ^1^Medical Sciences Research Center, ^2^Department of Pharmacy, The Second Affiliated Hospital of Southeast University, Nanjing 210003, China; ^3^Nanjing Longyuan Natural Polyphenol Synthesis Institute, Nanjing 210042, China

**Keywords:** Ellagic acid (EA), cancer, mechanism

## Abstract

Cancer is a leading cause of death worldwide. Cancer treatments by chemotherapeutic agents, surgery, and radiation have not been highly effective in reducing the incidence of cancers and increasing the survival rate of cancer patients. In recent years, plant-derived compounds have attracted considerable attention as alternative cancer remedies for enhancing cancer prevention and treatment because of their low toxicities, low costs, and low side effects. Ellagic acid (EA) is a natural phenolic constituent. Recent *in vitro* and *in vivo* experiments have revealed that EA elicits anticarcinogenic effects by inhibiting tumor cell proliferation, inducing apoptosis, breaking DNA binding to carcinogens, blocking virus infection, and disturbing inflammation, angiogenesis, and drug-resistance processes required for tumor growth and metastasis. This review enumerates the anticarcinogenic actions and mechanisms of EA. It also discusses future directions on the applications of EA.

## Introduction

Cancer is a leading cause of death in developed and developing countries^1^^,^^2^. Searching for new anticancer agents from plant sources is a realistic and promising approach which may lead to the discovery of many novel anti-cancer drugs^3^^,^^4^.

Ellagic acid (EA) is a naturally occurring phenolic constituent that is contained in ellagitannins in grapes, nuts, strawberries, black currents, raspberries, green tea, pomegranates, and the stem and bark of *Eucalyptus globulus*, *Eucalyptus maculata*, and nuts^5^. The International Union of Pure and Applied Chemistry name of EA is 2,3,7,8-tetrahydroxy-chromeno[5,4,3-cde]chromene-5,10-dione. The total EA quantities of different fruits and plants are provided in **Table 1**. EA exerts potent preventive and therapeutic effects against several types of cancers, including colon cancer, breast cancer, prostate cancer, skin cancer, esophageal cancer, and osteogenic sarcoma^14^^,^^15^. The anticarcinogenic properties of EA have drawn increasing attention globally.

**Table 1:** Total EA concentration of different plants

Fruits and plants	Total EA	Reference
Bananas	2*	6,7
Pear	4*	6,7
Tangerine	4*	6,7
Pineapples	6*	6,7
Plum	7*	6,7
Strawberry	31-78*	6-9
Pecan nut	33*	8
Walnut	59*	8
Raspberry	>150*	7,9
Cloudberry	>160*	7,9
Arctic Bramble	>160*	7,9
Strawberry cultivar	6-34.1**	10
Pongamia pinnata	1.5* (bark)	11
	0.1* (leaves)	
	0.4* (seeds)	
Geraniaceae	397*	12
Muscadine grape cultivars	587-1900* (skin)	13

*, mg/100 g dry weight; **, mg/100 g frozen weight.

## Anticarcinogenic effects and mechanisms of EA

### Antiproliferative and pro-apoptotic actions and their effects on subcellular signaling pathways

EA has *in vitro* and *in vivo* cancer chemopreventive properties. EA treatment is a new and highly effective strategy in reducing cancer carcinogenesis^16^. EA exerts anticancer effects through its antiproliferative and pro-apoptotic actions, as well as their effects on subcellular signaling pathways.

Han *et al*.^17^ observed that EA significantly reduces the proliferation and induces the apoptosis of human osteogenic sarcoma (HOS) cells as evidenced by chromosomal DNA degradation and apoptotic body appearance. EA progressively decreased the relative proliferation of the HOS cells in a dose-dependent manner, with an IC_50_ value of 6.5 µg/mL. Aside from the progressive, time- and dose-dependent increase in chromosomal DNA degradation, an increase in hypodiploid DNA content and significant time-dependent nuclear fragmentation was also observed.

EA at high concentrations (10-50 mmol/L) can stimulate the apoptosis and completely inhibit the proliferation of the human pancreatic adenocarcinoma cell lines MIA PaCa-2 and PANC-1. EA can accomplish these effects by decreasing nuclear factor-kappa B (NF-κB) activity, thereby activating the mitochondrial death pathway, which is associated with loss of mitochondrial membrane potential (Δψm), cytochrome C release, and caspase-3 activation^18^.

A recent study has indicated that EA at low concentrations (0.5-3 μM) triggers the apoptosis and inhibits the proliferation of the human pancreatic cancer cell lines MIA PaCa-2 and HPAF-II cells. EA elicits similar effects on pancreatic stellate cells, the progenitors of pancreatic cancer desmoplasia. *In vivo* dietary EA alone can decrease the size and cellularity of a tumor in a subcutaneous xenograft mouse model of pancreatic cancer^19^. Another *in vivo* study indicated that EA can inhibit pancreatic cancer growth in Balb/C nude mice; this inhibitory effect of EA was associated with the suppression of cell proliferation, activation of caspase-3, and induction of poly (ADP-ribosyl) polymerase cleavage. EA can also inhibit the expression of Bcl-2, cyclin D1, CDK2, and CDK6 while induce the expression of the pro-apoptotic protein Bax in tumor tissues as compared with untreated control tissues^20^.

Furthermore, EA (10-100 μM) can inhibit the proliferation of ovarian carcinoma ES-2 and PA-1 cells in a dose- and time-dependent manner by arresting both cell lines at the G_1_ phase. EA can accomplish these effects by increasing the expression of p53 and Cip1/p21 and decreasing the expression of cyclins D1 and E. EA can also induce caspase-3-mediated apoptosis by increasing the Bax/Bcl-2 ratio, one of the major phenomena that regulate apoptosis, and restore anoikis in both cell lines^21^.

A previous study^22^ in the human nasopharyngeal carcinoma cell line (NPC-BM1) indicated that EA reduces cell viability. The apoptosis features showed that DNA fragmentation and increased caspase-3 activity are associated with Bcl-2 downregulation. Furthermore, treatment of NPC-BM1 cells can inhibit human telomerase reverse transcriptase and human telomerase-associated protein 1, thereby decreasing telomerase activity.

Vanella *et al*.^16^ studied the precise molecular mechanisms involved in EA-induced apoptosis in prostate cancer cells. EA produces antiproliferative effects by inhibiting the activation of mammalian target of rapamycin and reducing the intracellular levels of β-catenin in the LNCaP human prostatic cancer cell line. EA also increases the percentage of apoptotic cells by downregulating anti-apoptotic proteins and silencing information regulator 1, human antigen R, and heme oxygenase-1. Furthermore, EA modulates the expression of apoptosis-inducing factor and the activation of caspase-3. Finally, EA increases the expression of the tumor suppressor protein p21.

The protein kinase C (PKC) signaling pathway is critical to cell proliferation, and over activation leads to abnormal tumor growth. The anticarcinogenic action of EA was confirmed after being administered to Dalton’s lymphoma-bearing mice^23^. EA acts by downregulating PKC, NF-κB, and c-Myc while upregulating transforming growth factor-β1 (TGF-β1). Lymphoma prevention by EA is further supported by the decrease in cell proliferation, cell viability, and ascite fluid accumulation, as well as the increase in the life span of DL mice. A new study from the same research group suggested that EA induces cancer cell death by blocking energy metabolism^24^.

Breast cancer is the most commonly diagnosed cancer among women worldwide. Two receptor pathways, estrogen receptor and tyrosine kinase receptors, especially the epidermal growth factor receptor family, are drivers of cell proliferation. These pathways are crucial to the development of both primary and recurrent breast cancers. EA not only interacts with and alters the effects of these pathways but also induces cell death (apoptosis and autophagy) by influencing kinase signaling *in vitro* and *in vivo*^25^. Furthermore, these pathways may prevent mammary tumors by suppressing the levels of E2-metabolizing enzymes during early-phase E2 carcinogenesis^26^.

### Prevention of DNA damage generated by oxidative stress and carcinogens

Epidemiological studies and large-scale clinical prevention trials suggested that oxidative stress causes genetic instabilities and functions in the initiation of human cancer. Therefore, effective inhibition of endogenous oxidative DNA damage may be a useful prevention strategy^27^. EA has high effectiveness in preventing oxidative DNA damage both *in vitro* and *in vivo*.

EA is a naturally occurring broad spectrum antioxidant. The primary antioxidant mechanism of EA has been attributed to the direct scavenging of free radicals, nitrogen reactive species, and ROS, including hydroxyl radicals, peroxyl radicals, NO_2_ radicals, and peroxynitrite. Other potential protective mechanisms of EA include shielding of DNA from attack and subsequent mutation by its direct association with this macromolecule, inhibition of ROS production, and chelation of metal ions, such as copper^28^^,^^29^.

EA at low doses (1 μM) is substantially effective (nearly 50% inhibition) in preventing dopamine/Cu (II)-mediated oxidatively generated DNA damage^30^. In EA-treated Chinese hamster lung fibroblast (V79-4) cells, more than 75% of the DPPH radical was scavenged in concentrations of 0.8-100 μg/mL. EA at 4, 20, and 100 μg/mL can inhibit lipid peroxidation by 55%, 79%, and 88%, respectively (IC_50_ value <4.0 μg/mL). The activities of the antioxidant enzymes superoxide dismutase, catalase, and glutathione peroxidase were also significantly increased in the EA-treated V79-4 cells^17^.

The cytotoxic and antiproliferative activities of EA against cancer cells do not affect normal cell viability. That is, EA is selectively cytotoxic to carcinoma cells but not to normal cells. A recent study^31^ has indicated that EA has minimal pro-oxidant nature but significant antioxidant property.

Microarray analysis^32^ revealed that EA modulates several genes. Specifically, EA overexpresses genes involved in DNA repair, such as xeroderma pigmentosum group A complementing protein, DNA ligase III, and DNA excision repair protein, by threefold to eightfold. By contrast, EA downregulates mitogen-activated protein kinase and MAP kinase kinase, which are involved in key cell-signaling pathways.

EA as a chemopreventive agent inhibits carcinogen bioactivation, carcinogen-to-DNA binding, and cancer cell growth^33^. For example, the formation of O6-methylguanine (O6-meGua) adducts and their persistence are closely linked to esophageal tumor induction in rats. The detection of O6-meGua adducts in the DNA of normal esophageal tissue taken from esophageal cancer patients in China also substantiates the function of methylating nitrosamines in esophageal cancer development. N-nitrosomethylbenzylamine (NMBA) is a procarcinogen that requires metabolic activation to produce its carcinogenic effect. EA exhibits inhibitory effects on NMBA tumorigenesis in the Fischer 344 rat esophagus. When administered in a semi-purified diet at concentrations of 0.4 and 4 g/kg, EA can significantly reduce (21%-55%) the average number of NBMA-induced esophageal tumors after 20 and 27 weeks of the bioassay^34^^,^^35^. This inhibition is correlated with reductions in the formation of NMBA-induced O6-meGua adducts in esophageal DNA, suggesting that the berries influence NMBA metabolism and reduce DNA damage^36^.

### Prevention of cancer metastasis by inhibiting the angiogenesis, migration, and invasion of cancer cells

Tumor metastasis is a complex cascade that is accompanied by various physiological alterations involved in angiogenesis, matrix metalloproteinase (MMP) upregulation, and extracellular matrix degradation; tumor metastasis allows cancer cells to proliferate and invade blood or lymphatic system, thereby enhancing cancer cell invasion and worsening prognosis^37^.

Angiogenesis is critical to tumor progression and metastasis. EA demonstrates anti-angiogenic effects both *in vitro* and *in vivo*. Sartippour *et al*.^38^ revealed that an oral administration of pomegranate extract standardized to ellagitannin content to severe combined immunodeficient mice can decrease prostate cancer xenograft size, tumor vessel density, VEGF peptide levels, and hypoxia-inducible factor 1α expression.

EA also exerts anti-angiogenetic effects via the VEGFR-2 signaling pathway in breast cancer^15^. The structure-based interaction between EA and VEGFR-2 was analyzed. EA can form hydrogen bonds and aromatic interactions within the ATP-binding region of the VEGFR-2 kinase unit and thus significantly inhibit a series of VEGF-induced angiogenesis processes, including proliferation, migration, and tube formation of endothelial cells.

Huang *et al*.^39^ illustrated a detailed mechanism of EA in angiogenesis. EA demonstrates anti-angiogenic effects by inhibiting MMP-2 activity and secretion, as well as suppressing the tube formation and migration of vascular endothelial cells. Suppressed reversion-inducing cysteine-rich protein with Kazal motifs (RECK) expression was observed in numerous human tumors, including colorectal, breast, pancreas, gastric, hepatocellular, prostate, and non-small cell lung carcinoma. The key action of RECK is to downregulate MMP-2 activity. EA-induced RECK at both mRNA and protein levels associates with the decrease in MMP-2 secretion.

The EA treatment of PANC-1 xenografted mice can inhibit the expression of the markers of angiogenesis (COX-2, HIF1α, VEGF, VEGFR, IL-6, and IL-8) and metastasis (MMP-2 and MMP-9) in tumor tissues. In addition, EA can significantly inhibit phospho-Akt, Gli1, Gli2, Notch1, Notch3, and Hey1. EA can also reverse epithelial-to-mesenchymal transition by upregulating E-cadherin and downregulating Snail, MMP-2, and MMP-9. The data suggest that EA can inhibit pancreatic cancer growth, angiogenesis, and metastasis by suppressing the Akt, Shh, and Notch pathways^20^.

In recent studies, EA has shown anti-invasive effects on androgen-independent human (PC-3) and rat (PLS10) prostate cancer cell lines; it also decreases the secretion of MMP-2 from both cells^37^. The authors further verified that EA significantly reduces the proteolytic activity of collagenase/gelatinase secreted from the PLS-10 cell line. In addition, EA dose dependently inhibits collagenase IV activity. EA reportedly inhibits chemotaxis of the breast cancer cells to stromal cell-derived factor 1α (SDF1α), a chemokine that attracts breast cancer cells to the bone^40^. Wang *et al*.^41^ showed that EA can inhibit the growth of hormone-dependent and hormone-refractory prostate cancer cells and inhibit their migration and their chemotaxis toward SDF1α. Moreover, EA can increase the expression of cell adhesion genes and decrease the expression of genes involved in cell cycle control and cell migration. Furthermore, EA can increase several well-known tumor-suppression miRNAs, decrease several oncogenic miRNAs, and inhibit the chemokines receptor type 4/SDF1α chemotaxis axis.

The capability of EA to inhibit the invasion of breast and prostate cancer cells makes this compound a potent and effective treatment for cancer prevention.

### Prevention of cancer initiation and progression through anti-inflammation

Acute inflammation is a part of the defense response, whereas chronic inflammation can lead to hepatocellular carcinoma (HCC), prostate cancer, colon cancer, breast cancer, and other common forms of cancer. The link between inflammation and cancer is tight. HCC is an inflammation-related cancer^42^ because the chronic inflammatory state is necessary for the initiation and development of liver cancer. Several studies have shown that chronic infections with hepatitis B virus (HBV) and hepatitis C virus (HCV) are major risk factors for HCC development^43^^-^^45^. Chronic inflammation also affects many cellular pathways, leading to fibrosis and cirrhosis and finally hepatocarcinogenesis. Colon cancer is another clear example of the tight link between inflammation and cancer. Inflammatory bowel disease ranks among the top three high-risk conditions for colon cancer. The risk for colorectal cancer increases with the duration and extent of the disease, confirming the active function of inflammation in cancer development. The regular use of nonsteroidal anti-inflammatory drugs also lowers the mortality from sporadic colon cancer and results in the regression of adenomas in familial adenomatous polyposis patients^46^.

Several pro-inflammatory gene products are crucial in suppressing apoptosis, proliferation, angiogenesis, invasion, and metastasis. Among these gene products are TNF and members of its super family, including IL-1α, IL-1β, IL-6, IL-8, IL-18, chemokines, MMP-9, VEGF, COX-2, and 5-LOX. The expression levels these genes are principally regulated by the transcription factor NF-κB. NF-κB mediates innate and adaptive immunity by initiating an inflammatory response to pro-inflammatory signals. NF-κB is constitutively active in most tumors and is induced by carcinogens, tumor promoters, carcinogenic viral proteins (HIV-tat, HIV-nef, HIV-vpr, KHSV, EBV-LMP1, HTLV1-tax, HPV, HCV, and HBV), chemotherapeutic agents, and gamma-irradiation. The persistent activation of NF-κB in tumor cells alters their ability to grow and differentiate. One of the best studied consequences of NF-κB activation is the enhanced survival of cancer cells. The role of persistent inflammation in aiding tumor development has led to the NF-κB family of transcription factors being strongly implicated in promoting cancer^47^^,^^48^. Anti-inflammatory agents that suppress NF-κB or NF-κB-regulated products should have a potential in the prevention and treatment of cancer^49^.

EA reportedly possesses anti-inflammatory properties. Khan *et al*.^50^ explored the possible chemopreventive mechanism of EA against NF-κB using the 3-D structure and X-ray crystallographic structure of the molecules, as well as molecular docking simulation software. They found that EA shows significant binding affinity with the Rel homology domain of the NF-κB precursor protein p105 with a binding energy of −7.99 Kcal and an inhibition constant of 1.38 μM. However, EA is not as effective as quercetin and 1-caffeoylquinic acid in inhibiting the target molecule. Rocha *et al*.^40^ discovered that pomegranate juice or a combination containing EA constituent increases breast cancer cell adhesion and decreases cancer cell migration. They also found that pro-inflammatory cytokines/chemokines are significantly reduced by these treatments. Therefore, these treatments have the potential to decrease inflammation and inhibit cancer progression.

Umesalma *et al*.^51^ investigated the effect of EA on Wistar albino rats with 1,2-dimethylhydrazine-induced colon cancer. They found that EA demonstrates anti-inflammatory property by downregulating inducible nitric oxide synthase, COX-2, TNF-α, and IL-6 through the inhibition of NF-κB, which is a prompter of tumorigenesis. They also found that EA exerts chemopreventive effects on colon carcinogenesis. Treatment with EA reduces both TGF-β and IL-6 levels in the LNCaP human prostatic cancer cells^16^.

The major functions and effective dose of EA on different cancer types are summarized in **Table 2**.

**Table 2:** Effects of EA in different cancer cell lines or xenografted animals

Cancer type	Cell line/animal	Effective concentration	Biological effects	Reference
Breast cancer	Cell line: MDA-MB-231	2.5-20 μM	Inhibits cancer cell proliferation and migration by downregulating VEGF-induced angiogenesis, VEGF-2 tyrosine kinase activity and its downstream MAPK, and PI3K/Akt pathways	15
	Animal: Female ACI rats	400 ppm	Downregulates 17β estradiol by reducing 17β-hydroxysteroid dehyrdogenase and reduces mammary tumor incidence	16
Osteogenic sarcoma	Cell line: ATCC CRL1343	4-100 µg/mL (IC_50_ =6.5 µg/mL)	Induces apoptosis by upregulating Bax and activating caspase-3	17
Pancreatic cancer	Cell line: MIA PaCa-2, and PANC-1	10-50 mM	Stimulates the mitochondrial pathway of apoptosis associated with mitochondrial depolarization, cytochrome C release, and downstream caspase activation	18
Ovarian carcinoma	Cell line: ES-2 and PA-1	10-100 μM	Elevates p53 and Cip1/p21, decreases cyclin D1 and E levels, and induces caspase-3-mediated apoptosis by increasing the Bax/Bcl-2 ratio	21
Nasopharyng- eal carcinoma	Cell line: NPC-BM1	50-200 μM	Reduces cancer cell viability by increasing caspase-3 activity, downregulating Bcl-2, and decreasing telomerase activity	22
Lymphoma	Animal: Dalton’s Lymphoma bearing mice	40-80 mg/kg body weight	Prevents cancer progression and increases life span of DL mice by downregulating the PKC signaling pathway and induces cancer cell death by blocking energy metabolism	23,24
Prostate cancer	Cell line: PLS10(rat)	80-200 μM (IC_50_ =100 µM)	Inhibits invasive potential through action on the activity of proteases, such as collagenase/gelatinase and collagenase IV	37

## Other indirect mechanisms involved in EA anticancer actions

### Radiosensitizing and counter radioresistance actions

Girdhani *et al*.^52^ investigated the mechanism of action of different anticancer and antioxidant agents, including EA, on both normal and cancer cells to develop effective protocols in practical radioprotection and cancer radiotherapy. They found that EA presents cytotoxic effects involving oxidative damage, membrane alteration, and damage to nucleic acid when combined with ionizing radiation in tumor cell lines. Further research^53^ indicated that EA can suppress the radiation-induced activation of receptor tyrosine kinases and NF-κB signaling, can modify cell survival and DNA repair efficacy, and may potentiate ceramide signaling. The radiosensitizing and counter radioresistance mechanisms of EA may provide a new approach to develop an effective treatment for cancer.

### Antivirus and liver-/heart-protective actions

The incidence of HCC continues to increase globally. Chronic HBV infections progress through stages of increasing inflammation associated with fibrosis and thus result in cirrhosis, which predisposes individuals to HCC. EA blocks the HBV-antigen secretion in HBV-infected hepatocytes and ameliorates the immune tolerance caused by HBeAg during HBV infection in HBeAg-Tg mice without adverse reactions^54^^,^^55^. EA also exhibits antifibrotic activity in the liver tissue of rats with CCl_4_-induced liver fibrosis. This result suggests that EA can be used as a therapy for HBV infection and thus reduce the risk for liver tumorigenesis.

EA also shows protective effects on liver and heart toxicity induced by cisplatin. Therefore, EA may be used in combination with cisplatin in cancer chemotherapy to improve cisplatin-induced oxidative stress parameters^56^.

### Inhibiting glutathione S-transferase (GST)-induced drug resistance

GST is a multifunctional detoxification protein that catalyzes the conjugation of glutathione to chemical toxins. GST overexpression in cancer confers resistance to chemotherapeutic agents. Inhibiting GST overexpression has been suggested as an approach to combat GST-induced resistance. EA inactivates the GSTs M1-1, M2-2, and P1-1 *in vitro* in a time- and concentration-dependent manner. This finding suggests that EA can be used as anticancer and chemopreventive agents because of their functions as chemomodulators in GST overexpression in malignancies^57^.

## Current problems and future directions

EA can act through multiple pathways and can be used as a dietary agent for preventing and treating many common forms of cancer. Through the action of human colonic microflora, EA is partially converted into metabolites, including hydroxy-6H-benzopyran-6-one derivatives, primarily urolithin A (UA); then, EA and urolithins enter the circulation^58^. Recent studies based on *in vitro* testing have shown preliminary evidence on the anti-inflammatory, anticarcinogenic, antiglycative, antioxidative, and antimicrobial effects of urolithins. Although the number of *in vivo* studies is still limited, their findings on the preventive effects of urolithins on gut and systemic inflammation encourage further researches^59^^,^^60^. However, the bioavailability of EA and urolithins is very low. Poor absorption from the gut, rapid metabolism, and lack of transport to the target organs may limit the bioavailability and clinical usefulness of EA and urolithins upon oral administration^61^. Furthermore, ABC transporters and Phase-II metabolism are involved in cancer cells as a mechanism of cancer resistance against urolithins through their conversion into glucuronide conjugates, which exert low antiproliferative activity^60^.

To overcome the bioavailability issues, many studies developed drug delivery systems, such as chitosan–glycerol phosphate (C-GP) in situ gelling system for the sustained subcutaneous delivery of EA^62^, EA-loaded poly (d,l-lactide-co-glycolide) nanoparticles for oral administration^63^, and using a new pH-sensitive polymer [Eudragit P-4135F (P-4135F)] to deliver EA to the lower small intestine in rats^64^. An increasing number of nanoparticles, liposomes, microemulsions, and polymeric implantable devices are emerging as viable alternatives for delivering therapeutic concentrations of EA into the systemic circulation. The results indicate that the bioavailability of EA has improved^65^.

EA and its metabolites have preventive and therapeutic potential against human cancers, and advanced drug delivery systems have potential for enhanced bioavailability. However, chemical modifications or more formulations that can bypass their poor oral bioavailability and eliminate hepatic first pass metabolism without compromising patient acceptability must be developed^66^. Mechanisms to maintain effective therapeutic concentrations in the blood due to the rapid drug metabolism should also be considered.
